# Does long-term fire suppression impact leaf litter breakdown and aquatic invertebrate colonization in pine flatwoods wetlands?

**DOI:** 10.7717/peerj.12534

**Published:** 2021-11-29

**Authors:** Houston C. Chandler, J. Checo Colón-Gaud, Thomas A. Gorman, Khalil Carson, Carola A. Haas

**Affiliations:** 1 Department of Fish and Wildlife Conservation, Virginia Polytechnic Institute and State University (Virginia Tech), Blacksburg, VA, United States of America; 2 The Orianne Society, Tiger, GA, United States of America; 3 Department of Biology, Georgia Southern University, Statesboro, GA, United States of America; 4 Aquatic Resources Division, Washington State Department of Natural Resources, Olympia, WA, United States of America; 5 Biological and Environmental Sciences Department, Troy University, Troy, AL, United States of America

**Keywords:** *Ambystoma bishopi*, Ephemeral wetlands, Reticulated Flatwoods Salamander, Leaf packs, Longleaf Pine, Wiregrass

## Abstract

Ephemeral wetlands are commonly embedded within pine uplands of the southeastern United States. These wetlands support diverse communities but have often been degraded by a lack of growing-season fires that historically maintained the vegetation structure. In the absence of fire, wetlands develop a dense mid-story of woody vegetation that increases canopy cover and decreases the amount of herbaceous vegetation. To understand how reduced fire frequency impacts wetland processes, we measured leaf litter breakdown rates and invertebrate communities using three common plant species (Longleaf Pine (*Pinus palustris*), Pineland Threeawn Grass (*Aristida stricta*), and Black Gum (*Nyssa sylvatica*)) that occur in pine flatwoods wetlands located on Eglin Air Force Base, Florida. We also tested whether or not the overall habitat type within a wetland (fire maintained or fire suppressed) affected these processes. We placed leaf packs containing 15.0 g of dried leaf litter from each species in both fire-maintained and fire-suppressed sections of three wetlands, removing them after 103–104 days submerged in the wetland. The amount of leaf litter remaining at the end of the study varied across species (*N. sylvatica* = 7.97 ± 0.17 g, *A. stricta* = 11.84 ± 0.06 g, and *P. palustris* = 11.37 ± 0.07 g (mean ± SE)) and was greater in fire-maintained habitat (leaf type: *F*_2,45_ = 437.2, *P* < 0.001; habitat type: *F*_1,45_ = 4.6, *P* = 0.037). We identified an average of 260 ± 33.5 (SE) invertebrates per leaf pack (range: 19–1,283), and the most abundant taxonomic groups were Cladocera, Isopoda, Acariformes, and Diptera. Invertebrate relative abundance varied significantly among litter species (approximately 39.9 ± 9.4 invertebrates per gram of leaf litter remaining in *N. sylvatica* leaf packs, 27.2 ± 5.3 invertebrates per gram of *A. stricta*, and 14.6 ± 3.1 invertebrates per gram of *P. palustris* (mean ± SE)) but not habitat type. However, both habitat (*pseudo*-F_1,49_ = 4.30, *P* = 0.003) and leaf litter type (*pseudo*-F_2,49_ = 3.62, *P* = 0.001) had a significant effect on invertebrate community composition. Finally, this work was part of ongoing projects focusing on the conservation of the critically imperiled Reticulated Flatwoods Salamander (*Ambystoma bishopi*), which breeds exclusively in pine flatwoods wetlands, and we examined the results as they relate to potential prey items for larval flatwoods salamanders. Overall, our results suggest that the vegetation changes associated with a lack of growing-season fires can impact both invertebrate communities and leaf litter breakdown.

## Introduction

Natural disturbances, events that disrupt an ecosystem or change the physical environment, occur across a variety of both spatial and temporal scales and have historically played a critical role in shaping many ecosystems (White & Pickett, 1985; White & Jentsch, 2001; Turner 2010). However, anthropogenic activity has caused significant changes to individual disturbance events and to overall disturbance regimes (Johnstone et al., 2016; Newman, 2019). These changes are important because disturbances can promote biodiversity and habitat heterogeneity that would otherwise be lost (Carlson et al., 1993; Townsend & Scarsbrook, 1997; Conway, Nadeau & Piest, 2010). Thus, restoring ecologically important disturbances are often the target of active management programs that attempt to emulate natural processes that have been disrupted by anthropogenic activity (Long, 2009).

Wildfire is a widespread natural disturbance, impacting a wide variety of terrestrial and aquatic ecosystems (Morgan et al., 2001; Bisson et al., 2003; Mouillot & Field, 2005; Butz, 2009). In the southeastern United States, the Longleaf Pine (*Pinus palustris*) ecosystem historically covered an area of approximately 35 million hectares (Frost, 1993). Longleaf Pine forests are a classic example of a fire-adapted ecosystem, experiencing frequent low-intensity fires (Henderson, 2006; Stambaugh, Guyette & Marshall, 2011). These regular fires historically maintained vegetation structure (*e.g.*, a pine overstory with thick herbaceous vegetation on the forest floor) and reduced leaf litter and woody debris build up (Brockway & Lewis, 1997). In the absence of natural or prescribed fires, Longleaf Pine forests transition to closed-canopy systems with abundant hardwoods in the mid-story and decreased diversity and abundance of herbaceous vegetation on the forest floor (Gilliam & Platt, 1999; Glitzenstein, Streng & Wade, 2003).

Ephemeral wetlands are a common landscape feature in Longleaf Pine forests and are characterized by a regular wetting and drying cycle that is tied to annual variation in precipitation and evapotranspiration rates. These wetlands are frequently geographically isolated (*i.e.,* lacking a consistent surface water connection to other water bodies; (Tiner, 2003; Cohen et al., 2016) and commonly support abundant and diverse biotic communities that are dependent on relatively predictable periods of inundation (*e.g.*, Golladay, Taylor & Palik, 1997; Kirkman et al., 1999; Erwin et al., 2016). Furthermore, these wetlands are subject to vegetation shifts similar to other Longleaf Pine ecosystems that can occur both from fire suppression and from poorly timed (*e.g.*, during the winter months when wetlands are more likely to have standing water present) prescribed fires (Kirkman, 1995; Bishop & Haas, 2005). Vegetation shifts change aquatic systems by altering leaf litter inputs, reducing the amount of structure available in the aquatic environment, and impacting other ecosystem processes (Mulhouse et al., 2005; Hinman & Brewer, 2007). Ultimately, these types of changes can impact the composition and abundance of aquatic communities (Hornung & Foote, 2006; Chandler et al., 2015).

Aquatic invertebrate communities are a critical component of ephemeral wetlands (Batzer & Wissinger, 1996; McInerney et al., 2017), functioning across multiple trophic levels by acting as both a prey base for other species and as predators in generally fishless environments (Murkin & Wrubleski, 1988; Batzer & Wissinger, 1996). Ephemeral wetlands can also support higher aquatic invertebrate biomass and differing community composition when compared to permanent wetlands with fish populations (Zimmer et al., 2001; McInerney et al., 2017). One of the important roles that invertebrates play in wetland ecosystems is contributing to leaf litter breakdown by consuming and physically breaking down leaf litter that falls into the wetland basin (Fazi & Rossi, 2000; Gingerich, Panaccione & Anderson, 2015). Breakdown rates can vary widely across different leaf litter species (Leroy & Marks, 2006), and litter inputs into the wetland can broadly impact both biotic and abiotic processes (Stoler & Relyea, 2016). The structure, nutrient content, and availability of individual leaf litter species can all impact the invertebrate community, with effects potentially transitioning to higher trophic levels (Batzer & Palik, 2007; Stoler & Relyea, 2016).

Here, we describe the results of a field experiment testing the effects of habitat type (*i.e.,* fire maintained *vs.* fire suppressed) on leaf litter breakdown and invertebrate communities in pine flatwoods wetlands. We assessed these environmental processes for three species of leaf litter: Longleaf Pine (*P. palustris*), Pineland Threeawn Grass, commonly referred to as wiregrass, (*Aristida stricta*), and Black Gum (*Nyssa sylvatica*). *Pinus palustris* and *A. stricta* are commonly found in fire-maintained wetlands, while *N. sylvatica* is mostly restricted to wetlands with reduced fire frequency or deeper portions of wetlands that are less likely to experience regular fires (Chandler, 2015). We predicted that fire-maintained sections of wetlands would support more abundant invertebrate communities, leading to higher breakdown rates. Furthermore, we predicted that *P. palustris* and *A. stricta* would breakdown more slowly than *N. sylvatica* because of lower surface area for invertebrate colonization. Finally, we assessed invertebrate communities overall and specifically focused on taxa that are important food sources for larval Reticulated Flatwoods Salamanders (*Ambystoma bishopi*), a US Federally endangered species (U. S. Fish and Wildlife Service, 2009). Larval flatwoods salamanders feed primarily on aquatic invertebrates (*e.g.*, Isopoda, Amphipoda, and Copepoda) and, while little is known about foraging behavior, opportunistic observations suggest that larvae forage in and around herbaceous vegetation as well as along benthic substrates (Palis, 1996; Sekerak, Tanner & Palis, 1996; Whiles et al., 2004).

## Materials & Methods

### Study sites

All field work was conducted on Eglin Air Force Base (Eglin) and access to field sites was approved by the US Fish and Wildlife Service and Jackson Guard (Eglin’s Natural Resources Division; Cooperative Agreement Number F14AC00068). Eglin is a large military installation covering over 188,459 ha of the Florida Panhandle’s Gulf Coastal Plain. Consisting largely of sandhills and other upland habitat, Eglin also contains some of the best remaining examples of mesic pine flatwoods. These forests have sandy, poorly drained soils and ephemeral, geographically isolated wetlands embedded within the surrounding pine forest. Eglin has an extensive active habitat management program that routinely applies prescribed fire to pine forests across the installation. However, there have been persistent challenges associated with burning inside of wetland basins, and most pine flatwoods wetlands on the installation were either partially or completely degraded by long-term fire suppression and exclusion. Pine flatwoods wetlands on Eglin support diverse amphibian communities, including breeding populations of Reticulated Flatwoods Salamanders (U. S. Fish and Wildlife Service, 2009). During their aquatic larval phase, flatwoods salamanders depredate a variety of freshwater invertebrate groups (Whiles et al., 2004), serving as important predators in the wetlands that they inhabit.

### Study design

We collected leaf litter from the three species of interest (*P. palustris*, *A. stricta*, and *N. sylvatica*) in areas surrounding pine flatwoods wetlands on Eglin. We raked *P. palustris* needles from underneath large trees, collected *N. sylvatica* leaves from plants that had been manually removed from wetlands, and collected *A. stricta* material from standing dead stems at the end of the reproductive period. We dried all leaf litter at room temperature for approximately one week. We then filled coarse mesh bags (8-mm openings), allowing for invertebrate colonization, with 15.0 g of dried leaf litter from a single species.

We placed a total of 72 leaf packs across three pine flatwoods wetlands on Eglin that were part of long-term monitoring projects focused on wetland communities and management (*e.g.*, Gorman, Haas & Himes, 2013). We selected wetlands that had areas of both high (fire maintained) and low (fire suppressed) herbaceous vegetation cover. We note here that we chose wetlands based on their vegetation characteristics and not on a specific fire history. All wetlands are located in actively managed pine flatwoods on Eglin with similar overall management histories. Based on limited data, wetland basins included in this study experienced 2–3 prescribed fires from 2012–2015, and we documented fire effects 86% of the time in fire-maintained sections and 29% of the time in fire-suppressed sections (based on leaf pack locations). Furthermore, current conditions are the result of multiple management activities, including both prescribed fire and mechanical removal of woody vegetation (Gorman, Haas & Himes, 2013).

In each wetland, we deployed leaf packs along 10-m transects in both habitat types, placing one leaf pack of each plant species at both ends and in the middle of the transect (9 per transect and 18 per wetland). We separated individual leaf packs by approximately 30 cm perpendicular to the transect. We added leaf packs to wetlands on 10 and 11 November 2015 and secured them to the bottom of the wetland using metal gardening stakes. Finally, in one wetland, we doubled the number of leaf packs to examine the effects of time submerged on breakdown and invertebrate communities (logistical constraints prevented this effort in all wetlands). We positioned these leaf packs adjacent to the same transect, placing leaf packs containing the same plant species next to one another.

Wetlands remained flooded for the entire duration of the study, and we removed leaf packs at two time periods. First, we collected the leaf packs from the additional replicate in the single wetland on 5 December 2015 (25 days submerged). All other leaf packs were removed from wetlands on 22 February 2016 (103–104 days submerged). Upon removal from the wetland, we immediately placed all leaf packs in 95% ethanol to stop further decomposition of leaf material and to preserve invertebrates until samples could be processed.

We washed all of the remaining leaf litter to separate out invertebrates using a 125 µm sieve. All remaining material from the leaf packs was then dried at 55 °C for at least 48 h. We then weighed this material to obtain dry mass (DM). Next, we ground samples into a fine powder by milling at 25,000 rpm for either 90 s (*N. sylvatica*) or 180 s (*P. palustris* and *A. stricta*) (IKA^®^ Tube Mill 100 control). We burned ground samples in a muffle furnace at 500 °C for 1 h to obtain ash-free dry mass (AFDM). For the 25-day replicate, we burned the entire sample, while we burned approximately 1 g of the 103–104-day samples and calculated the AFDM from this subsample. We used the AFDM of each sample to calculate the processing coefficient (k) using the exponential decay model (Petersen & Cummins, 1974; Maloney & Lamberti, 1995; Benfield, Fritz & Tiegs, 2017). We used the estimated processing coefficients to calculate number of days it would take for 99% of the sample to breakdown. Finally, we identified all invertebrates to broad taxonomic groups that were easily identifiable (order in most cases).

In addition to leaf packs, we also quantified herbaceous vegetation cover and canopy cover as part of other ongoing projects. Herbaceous vegetation and canopy cover were measured at points along each wetland’s longest axis, partially overlapping the locations with leaf packs (Gorman, Haas & Himes, 2013). We estimated the percent herbaceous vegetation cover using a 0.5 m × 0.2 m Daubenmire frame and the Daubenmire cover class scale (Daubenmire, 1959). We measured canopy cover by averaging the canopy cover recorded in each of the four cardinal directions with a convex spherical densiometer. All vegetation data were collected during the fall of 2014, and for this assessment, we included the two sampling locations that were closest to the leaf packs.

### Statistical analyses

Prior to all analyses, we excluded all results from one 104-day leaf pack (*A. stricta*, fire-suppressed habitat) and the invertebrate results from one 25-day leaf pack (*P. palustris*, fire-maintained habitat) because these data were not consistent with the other results. Furthermore, we only included data from leaf packs submerged for 103–104 days in the following analyses. We tested for an effect of leaf litter species and habitat type (fire suppressed or maintained) on leaf litter breakdown and invertebrate abundance using a series of linear mixed effects models (LMM). First, we fit a mixed effects model to test for the effects of litter species and habitat type on the amount of leaf litter remaining at the end of the experiment. We treated the wetland as a random effect to account for non-independence in the leaf packs collected from the same wetland. Leaf litter species, habitat type, and their interaction were included in the model as fixed effects. Second, we fit two similar models using the total invertebrate abundance and the abundance of groups that make up the primary components of the larval flatwoods salamander diet. We defined these groups as Isopoda, Amphipoda, and Copepoda, which make up approximately 65% of prey items found in larval flatwoods salamander stomachs (Whiles et al., 2004). Both measures of invertebrate abundance were standardized by the mass remaining in their respective leaf pack prior to analyses. Invertebrate models also contained leaf species, habitat type, and their interaction as fixed effects and wetland as a random effect. We verified that the assumptions of linearity, normality, and homogeneity of the residuals were met in all three models using diagnostic plots. Finally, we performed pairwise comparisons using Tukey’s HSD when tests on main effects indicated a significant difference among leaf litter species.

To further examine the composition of invertebrate communities, we visualized the community data using a non-metric multidimensional scaling (NMDS) plot. We graphed community data using Bray–Curtis dissimilarities and verified that the Stress statistic for the NMDS was less than 0.2 (Clarke, 1993). We tested for differences in community composition among leaf litter species and habitat type using a distance-based permutational multi-variate analysis of variance (PERMANOVA; Anderson & Walsh, 2013). We conducted the PERMANOVA using Bray–Curtis dissimilarity indices and 999 permutations. The individual wetland was treated as a block in this analysis, and we tested for significance using the marginal effects. We also tested for differences in group dispersions (variance) following Anderson (2006). All statistical analyses were performed in R (R Core Team, 2020), and NMDS, PERMANOVA, and tests for dispersion were available in the *vegan* package (Oksanen et al., 2019). Mixed models were fit using the *lme4* package (Bates, Maechler & Walker, 2015), and pairwise comparisons were performed using the *emmeans* package (Lenth, 2021).

## Results

**Figure 1:**
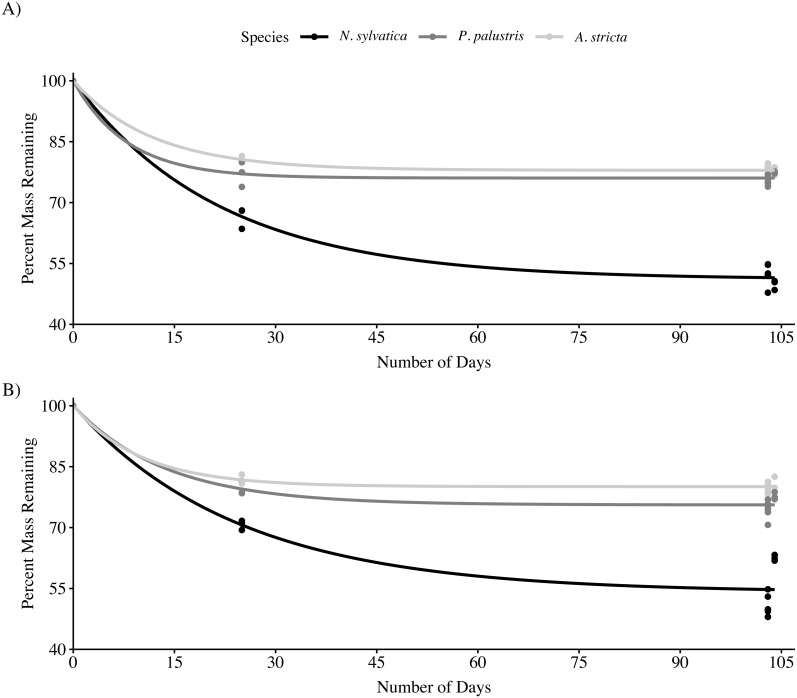
Mass loss for Longleaf Pine (*Pinus palustris*), Pineland Threeawn Grass (*Aristida stricta*), and Black Gum (*Nyssa sylvatica*) measured in three pine flatwoods wetlands on Eglin Air Force Base. (A) Mass loss from fire-maintained habitats. (B) Mass loss from fire-suppressed habitats. Solid lines represent the non-linear least squares fit for each group, approximating the rate of mass loss over time using 25-day leaf packs collected in a single wetland.

Across all three leaf species and both habitat types, the rate of mass loss was highest during the first 25 days after being submerged (Fig. 1). After 103–104 days submerged, *N. sylvatica* had the fastest mean k rate (−0.0062 ± 0.0002 (SE)) followed by *P. palustris* (−0.0027 ± 0.00006 (SE)) and *A. stricta* (−0.0023 ± 0.00005 (SE)). There was no interaction effect between habitat and leaf type on the amount of leaf litter remaining after 103–104 days in the wetland (LMM: *F*_2,45_ = 2.0, *P* = 0.15). However, both habitat (LMM: *F*_1,45_ = 4.6, *P* = 0.037) and leaf type (LMM: *F*_2,45_ = 437.2, *P* < 0.001) significantly impacted leaf litter breakdown after 104 days (Fig. 1; Table 1, Table 2). *Nyssa sylvatica* (weight remaining = 7.97 ± 0.17 g (mean ± SE)) leaves broke down faster than both *P. palustris* (weight remaining = 11.37 ± 0.07 g (mean ± SE)) (*P* < 0.0001) and *A*. *stricta* (weight remaining: = 11.84 ± 0.06 g (mean ± SE)) (*P* < 0.0001). Breakdown was slower, on average, in fire-suppressed sections of wetlands. However, the effect was small compared to the differences between leaf types and was not consistent across wetlands (Table 1).

**Table 1:** Leaf litter breakdown metrics recorded in pine flatwoods wetlands on Eglin Air Force Base, Florida. Breakdown metrics for three plant species (Longleaf Pine (*Pinus palustris*), Pineland Threeawn Grass (*Aristida stricta*), and Black Gum (*Nyssa sylvatica*)) after 103–104 days submerged. Leaf packs were placed in sections of wetlands with vegetation characteristics indicative of fire-maintained and fire-suppressed habitat. Values represent means ± standard deviations from three leaf packs of each species. The processing coefficient (k) was estimated using an exponential decay model.

	% Litter remaining	*k*	Days to 99% decomposition
**Pond 53**			
Fire maintained			
*A. stricta*	0.78 ± 0.01	−0.0024 ± 0.0001	1898 ± 91
*P. palustris*	0.77 ± 0.004	−0.0025 ± 0.0001	1857 ± 37
*N. sylvatica*	0.50 ± 0.01	−0.0067 ± 0.0002	688 ± 24
Fire suppressed			
*A. stricta*	0.81 ± 0.02	−0.0020 ± 0.0002	2305 ± 261
*P. palustris*	0.78 ± 0.01	−0.0024 ± 0.0001	1906 ± 87
*N. sylvatica*	0.63 ± 0.01	−0.0045 ± 0.0001	1020 ± 24
**Pond 16**			
Fire maintained			
*A. stricta*	0.77 ± 0.02	−0.0025 ± 0.0003	1843 ± 187
*P. palustris*	0.76 ± 0.01	−0.0027 ± 0.0001	1728 ± 88
*N. sylvatica*	0.52 ± 0.04	−0.0064 ± 0.0007	721 ± 72
Fire suppressed			
*A. stricta*	0.80 ± 0.01	−0.0021 ± 0.0001	2174 ± 132
*P. palustris*	0.76 ± 0.01	−0.0027 ± 0.0002	1713 ± 97
*N. sylvatica*	0.51 ± 0.03	−0.0065 ± 0.0006	714 ± 65
**Pond 12**			
Fire maintained			
*A. stricta*	0.79 ± 0.01	−0.0023 ± 0.0001	2017 ± 75
*P. palustris*	0.75 ± 0.01	−0.0028 ± 0.0001	1645 ± 71
*N. sylvatica*	0.53 ± 0.02	−0.0062 ± 0.0003	749 ± 34
Fire suppressed			
*A. stricta*	0.79 ± 0.01	−0.0023 ± 0.0001	2022 ± 79
*P. palustris*	0.73 ± 0.02	−0.0030 ± 0.0003	1535 ± 159
*N. sylvatica*	0.50 ± 0.03	−0.0067 ± 0.0005	692 ± 51

**Table 2:** Parameter estimates for three linear mixed effects models. Linear mixed effects models estimated the effects of habitat type (fire suppressed or fire maintained) and leaf litter species (Longleaf Pine (*Pinus palustris*), Pineland Threeawn Grass (*Aristida stricta*), and Black Gum (*Nyssa sylvatica*)) on leaf litter breakdown, invertebrate relative abundance, and the relative abundance of invertebrate groups that are important prey items for larval flatwoods salamanders. Leaf litter breakdown and invertebrate communities were measured using leaf packs in three wetlands on Eglin Air Force Base, Florida.

Model	Habitat	Leaf species	Estimate	Standard error
Leaf litter breakdown	Fire suppressed	*N. sylvatica*	8.21	0.18
	Fire Suppressed	*P. palustris*	3.13	0.20
	Fire Suppressed	*A. stricta*	3.83	0.21
	Fire maintained	–	−0.48	0.20
Invertebrate relative abundance	Fire suppressed	*N. sylvatica*	31.53	10.17
	Fire suppressed	*P. palustris*	−18.12	12.70
	Fire suppressed	*A. stricta*	−4.14	13.10
	Fire maintained	–	16.73	12.70
Relative larval flatwoods	Fire suppressed	*N. sylvatica*	18.78	3.00
salamander prey abundance	Fire suppressed	*P. palustris*	−14.16	3.63
	Fire suppressed	*A. stricta*	−9.80	3.74
	Fire maintained	–	−2.82	3.63

**Figure 2:**
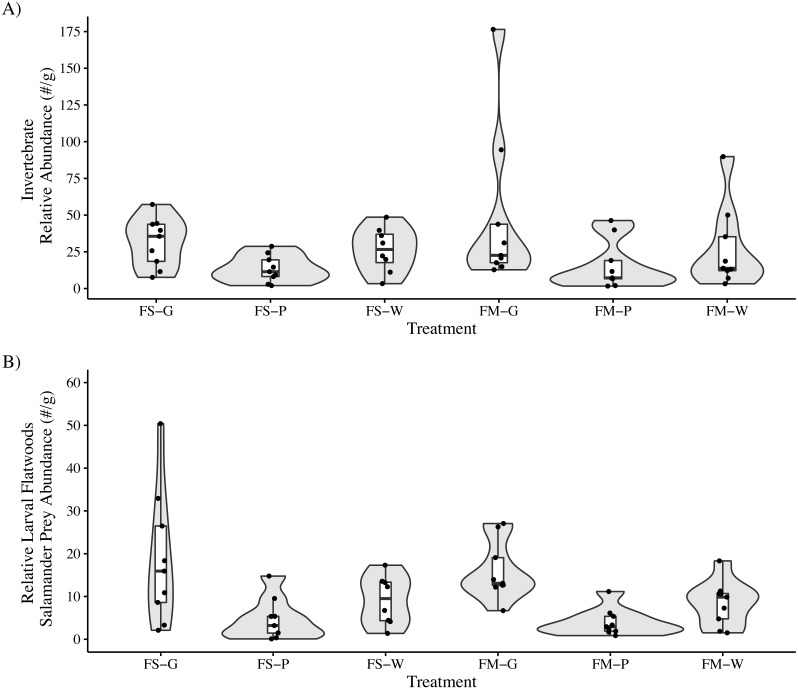
Invertebrate relative abundance measured in leaf packs in three pine flatwoods wetlands on Eglin Air Force Base, Florida. (A) Total relative invertebrate abundance. (B) Relative abundance of taxa (Isopoda, Amphipoda, and Copepoda) important to larval flatwoods salamander diets. Leaf packs represented one of six treatments that varied by leaf type and the overall habitat type in that portion of the wetland (FS-G: fire-suppressed, Black Gum (*Nyssa sylvatica*); FS-P: fire-suppressed, Longleaf Pine (*Pinus palustris*); FS-W: fire-suppressed, Pineland Threeawn Grass, commonly referred to as wiregrass, (*Aristida stricta*); FM-G: fire-maintained, *N. sylvatica*; FM-P: fire-maintained, *P. palustris*; FM-W: fire-maintained, *A. stricta*). Relative abundance is calculated as the number of invertebrates divided by the dry mass of remaining leaf litter, and polygons represent the mirrored kernel density plot, showing the smoothed distribution of the data points.

We identified a total of 2,677 invertebrates in leaf packs (mean: 158 per pack, range: 14–379, SE: 32.2) collected after 25 days submerged and 14,062 invertebrates in leaf packs (mean: 260 per pack, range: 19–1,283, SE: 33.5) collected after 103–104 days submerged. The most abundant invertebrate groups were Cladocera, Isopoda, Acariformes, and Diptera, accounting for approximately 93% of all invertebrates identified across all samples (Table 3). For leaf packs submerged 103–104 days, there was no interaction effect between litter species and habitat type on the total invertebrate relative abundance (LMM: *F*_2,45_ = 0.52, *P* = 0.60) or on the relative abundance of preferred flatwoods salamander prey items (LMM: *F*_2,45_ = 0.12, *P* = 0.88). Leaf type but not habitat type affected both overall invertebrate relative abundance (LMM: Leaf: *F*_2,45_ = 3.98, *P* = 0.03; Habitat: *F*_1,45_ = 0.70, *P* = 0.41) and the relative abundance of flatwoods salamander prey items (LMM: Leaf: *F*_2,45_ = 13.4, *P* = 0.00003; Habitat: *F*_1,45_ = 0.40, *P* = 0.53; Table 2). *Nyssa sylvatica* leaf packs (39.9 ± 9.4 (mean ± SE) invertebrates per gram of leaf litter remaining) had higher total invertebrate relative abundance than *P. palustris* (14.6 ± 3.1 (mean ± SE) invertebrates per gram of leaf litter remaining; Tukey’s HSD: t_45_ = 2.8, *P* = 0.007) but not *A. stricta* (27.2 ± 5.3 (mean ± SE) invertebrates per gram of leaf litter remaining; Tukey’s HSD: t_45_ = 1.4, *P* = 0.17). Similarly, *N. sylvatica* leaf packs (17.4 ± 2.8 (mean ± SE) invertebrates per gram of leaf litter remaining) had higher flatwoods salamander prey relative abundance than *P. palustris* (4.3 ± 0.9 (mean ± SE) invertebrates per gram of leaf litter remaining) and *A. stricta* (8.8 ± 1.3 (mean ± SE) invertebrates per gram of leaf litter remaining) (Tukey’s HSD: t_45_ = 5.1, *P* < 0.0001 and t_45_ = 3.3, *P* = 0.002, respectively) (Fig. 2).

**Table 3:** Invertebrate abundance in leaf packs that were placed in pine flatwoods wetlands on Eglin Air Force Base, Florida. Invertebrate groups collected in 54 leaf litter packs submerged for 103–104 days in ephemeral wetlands. Leaf packs containing either Longleaf Pine (*Pinus palustris*), Pineland Threeawn Grass (*Aristida stricta*), or Black Gum (*Nyssa sylvatica*) were placed in either fire-suppressed or fire-maintained habitat in three wetlands. Values represent the mean (±standard deviation) abundance of each invertebrate group averaged across nine replicates. Taxonomic groups marked with an asterisk were dominated by a single family but were grouped to higher taxonomic levels for all analyses (Isopoda - Asellidae, Amphipoda - Gammaridae, Diptera - Chironomidae, Hemiptera - Corixidae, Zygoptera - Coenagrionidae, Acariformes - Hydrachnidae, Gastropoda - Planorbidae).

	Fire suppressed			Fire maintained		
	Gum	Pine	Wiregrass	Gum	Pine	Wiregrass
Isopoda*	129.3 ± 113.3	41.4 ± 52.1	80.4 ± 70.6	105.0 ± 40.5	42.6 ± 36.1	91.7 ± 57.7
Amphipoda*	8.6 ± 4.0	9.4 ± 8.6	13.8 ± 26.6	12.6 ± 23.3	2.0 ± 2.7	5.4 ± 5.5
Anostraca	0.1 ± 0.3	—	—	—	—	—
Cladocera	23.0 ± 23.7	9.6 ± 7.3	62.1 ± 81.2	189.8 ± 329.1	64.0 ± 93.9	127.8 ± 182.0
Copepoda	7.7 ± 5.7	1.3 ± 1.4	5.7 ± 4.3	4.0 ± 4.2	1.0 ± 1.3	1.2 ± 1.7
Decapoda	—	—	—	0.2 ± 0.4	—	—
Diptera*	38.2 ± 13.7	24.7 ± 20.8	22.6 ± 17.9	12.3 ± 5.3	9.7 ± 11.2	12.1 ± 14.0
Hemiptera*	—	0.2 ± 0.4	—	—	0.1 ± 0.3	0.2 ± 0.4
Coleoptera	0.2 ± 0.4	0.1 ± 0.3	—	0.1 ± 0.3	0.1 ± 0.3	1.6 ± 2.3
Odonata	—	0.1 ± 0.3	—	—	—	—
Anisoptera	—	—	—	0.6 ± 1.3	0.2 ± 0.7	0.1 ± 0.3
Zygoptera*	—	—	—	1.0 ± 1.4	1.6 ± 2.4	2.7 ± 3.8
Acariformes*	38.3 ± 69.7	63.2 ± 96.7	122.8 ± 170.0	21.4 ± 25.2	56.8 ± 64.4	69.2 ± 67.9
Collembola	0.3 ± 0.7	0.0 ± 0.0	0.3 ± 1.0	0.2 ± 0.4	0.1 ± 0.3	—
Gastropoda*	1.2 ± 2.6	0.7 ± 1.3	0.1 ± 0.3	14.4 ± 20.3	2.8 ± 3.3	2.3 ± 4.2

**Figure 3:**
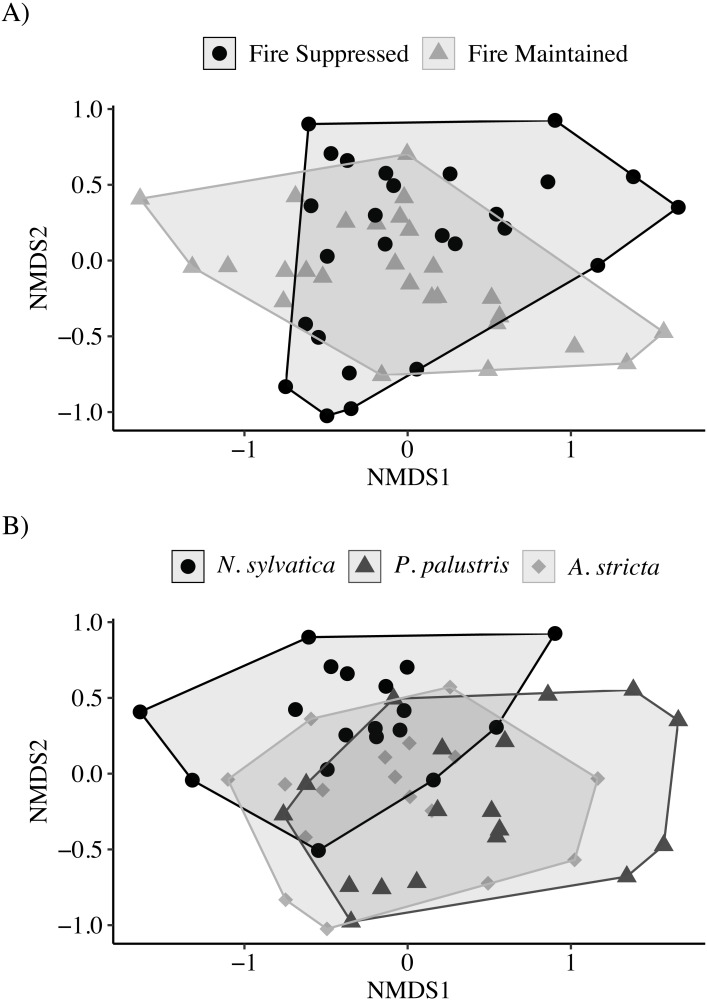
Non-metric Multidimensional Scaling (NMDS) plots of invertebrate communities measured in leaf packs from pine flatwoods wetlands on Eglin Air Force Base, Florida. (A) Leaf packs were placed in either fire-suppressed or fire-maintained habitat. (B) Leaf packs contained one of three leaf types: Longleaf Pine (*Pinus palustris*), Pineland Threeawn Grass (*Aristida stricta*), or Black Gum (*Nyssa sylvatica*).

The NMDS ordination showed only marginal separation among habitat types and larger separation among leaf types (Stress = 0.13; Fig. 3). The PERMANOVA indicated that both habitat (PERMANOVA: *pseudo*-*F*_1,49_ = 4.30, *P* = 0.003) and leaf litter type (PERMANOVA: *pseudo*-*F*_2,49_ = 3.62, *P* = 0.001) were significantly affecting invertebrate community composition. Similar to the above results, invertebrate communities observed on *N. sylvatica* had larger differences from those observed on *P. palustris* and *A. stricta* leaf litter, while the community observed on *P. palustris* almost completely overlapped the community observed on *A. stricta* (Fig. 3). The distance-based tests for homogeneity of community dispersions indicated that variation was similar across groups (Leaf: *pseudo*-*F*_2,50_ = 0.1.6, *P* = 0.23; Habitat: *pseudo*-*F*_1,51_ = 0.98, *P* = 0.34).

Environmental characteristics varied across the fire-maintained and fire-suppressed sections of wetlands. Herbaceous vegetation cover was nearly absent from fire-suppressed sections of the three wetlands (2.5 ± 0.0% in each wetland (mean ± SE)), while canopy cover showed more variation across the three wetlands (33–92%). In the fire-maintained habitat, herbaceous vegetation cover ranged from 63–85%, and canopy cover ranged from 8–16%.

## Discussion

The ecology of pine flatwoods wetlands is primarily regulated by wetland hydrology and fire regime. Both of these factors directly impact floral and faunal communities within wetlands along with a variety of biotic and abiotic processes that shape wetland ecosystems (Brockway & Lewis, 1997; Powell et al., 2005; Gorman, Haas & Bishop, 2009; Watts, 2013). Our results indicate that vegetation community composition within pine flatwoods wetlands impacts both leaf litter breakdown and aquatic invertebrate communities. Overall, leaf species had a larger effect on these processes than the broad vegetation characteristics within a wetland. However, in natural wetlands, leaf species and vegetation characteristics are fundamentally linked because certain tree and shrub species (*i.e.,* ones that increase canopy cover) are generally limited to either fire-suppressed sections of wetlands or the deepest areas in fire-maintained wetlands (Kirkman, 1995). Our results, along with other work, strongly indicate that altered fire regimes (particularly a loss of growing-season fire) fundamentally alter wetland ecosystems that are embedded within fire-dependent landscapes (De Szalay & Resh, 1997; Bixby et al., 2015; Chandler, Haas & Gorman, 2015).

Over the course of our study, a majority of the observed mass loss in all three leaf species occurred within 25 days of leaf packs being submerged. This rapid initial breakdown is generally driven by a combination of microbial activity and leaching of secondary compounds into the aquatic environment (Tietema & Wessel, 1993). After this initial period, *N. sylvatica* leaves continued to break down, while both *P. palustris* and *A. stricta* leaf packs lost little mass after the initial period. Over the entire study period, *N. sylvatica* leaf packs lost approximately 15–28% more mass than either *P. palustris* or *A. stricta*. The decay rates that we observed for *N. sylvatica* are similar to those previously reported (Battle & Golladay, 2001; Battle & Golladay, 2007). Interspecific differences in leaf litter breakdown can be attributed to the chemical make-up of the leaves (Battle & Golladay, 2007) and the abundance of invertebrates, particularly shredders, present (Tiegs et al., 2008), although the effects of invertebrates on leaf breakdown are variable across studies (Battle & Golladay, 2001; Fuell et al., 2013). Our results showed that *N. sylvatica* leaf packs contained more invertebrates relative to the amount of material remaining, possibly contributing to their continued breakdown across the entire 104-day period.

We also observed differences in breakdown rates between habitat types, although these effects were smaller overall and appeared to vary among the three wetlands. Leaf litter breakdown in ephemeral wetlands is driven by multiple processes that span both aquatic and terrestrial environments, such as water temperature, water chemistry, flooding regime (both depth and hydroperiod), and the above-mentioned biotic factors (Álvarez & Bécares, 2006; Battle & Golladay, 2007; Gingerich, Panaccione & Anderson, 2015). Canopy cover was variable across the three wetlands included in this study, potentially influencing the breakdown rate between habitat types in individual wetlands by impacting water temperatures (Werner & Glennemeier, 1999; Becker et al., 2012). This interpretation is supported by our observation that the largest within-wetland difference in breakdown rates occurred in the wetland with the highest canopy cover in the fire-suppressed section (*i.e.,* 92% in Pond 53 compared to just 62% and 33% in Ponds 12 and 16, respectively; Table 1). All fire suppressed sections of wetlands had almost no herbaceous vegetation but the variability in canopy cover may reflect differences in the severity of fire suppression or in the species composition of the mid-story vegetation (Peterson & Reich, 2008). While none of the wetlands experienced fire during our study, fires can impact water quality within wetlands, potentially altering breakdown rates (Battle & Golladay, 2003). Overall, it appears that differences between fire-suppressed and fire-maintained habitats may alter leaf breakdown rates in some cases, but that these effects are inconsistent and small compared to differences between leaf litter types.

Observed differences in invertebrate relative abundance were attributed to the different leaf litter species but not to the overall habitat type. Across both habitat types, *N. sylvatica* leaf packs tended to support more abundant invertebrate communities relative to the amount of leaf litter remaining at the end of the study. Higher invertebrate abundance may be related to differences in leaf structure for invertebrate colonization (*e.g.*, complex *N. sylvatica* leaves *vs.* cylindrical *P. palustris* needles and *A. stricta* stalks). Greater complexity and surface area can increase the abundance and diversity of invertebrate colonizers (Beckett, Aartila & Miller, 1992; Jeffries, 1993). Furthermore, leaf litter quality is often hypothesized to account for differences in invertebrate abundance across leaf species, and coniferous leaf litter has lower nutrient quality when compared to deciduous leaf litter (Polyakova & Billor, 2007; Hisabae, Sone & Inoue, 2010). However, coniferous leaf litter can be an important food resource for invertebrate communities when other litter types are scarce (Sakai et al., 2016), which may be the case in fire-maintained pine flatwoods wetlands where deciduous leaf litter is generally absent.

Our results indicated that there was little effect of overall habitat type on invertebrate relative abundance when sampling using leaf packs. These results starkly contrast some of our previous survey work in these wetlands that used dip net surveys to quantify invertebrate communities and showed a large difference across fire-maintained and fire-suppressed habitats (Chandler et al., 2015). These observed differences between studies are likely driven by the different sampling methodologies employed (*i.e.,* sampling in the water column *vs.* colonization in leaf packs). Ultimately, both leaf packs and dip net surveys over relatively small temporal and spatial scales are only targeting a subset of the aquatic invertebrate communities in these wetlands. In tandem, the results reported here and from Chandler et al. (2015) indicate that invertebrate abundance is likely similar throughout wetland habitat types but that both the composition and spatial arrangement (*i.e.,* in leaf litter *vs.* in standing herbaceous vegetation) of invertebrate communities varies across habitats.

Differences in the habitat use of invertebrate communities could have important implications for flatwoods salamanders because larvae are primarily found in areas with thick herbaceous vegetation (Sekerak, Tanner & Palis, 1996; Gorman, Haas & Bishop, 2009), and wetlands lacking sufficient area with dense herbaceous vegetation support few or no flatwoods salamanders (Brooks et al., 2019; Wendt et al., 2021). This association may be linked to egg deposition sites (Gorman et al., 2014) but could also reduce predation pressure, increase foraging opportunities for larvae, or both. Limited observations suggest that larval flatwoods salamanders forage in and around herbaceous vegetation and along edges of wetland basins (Palis, 1996; Sekerak, Tanner & Palis, 1996; Whiles et al., 2004), but larvae can be forced into deeper areas with high canopy cover and little herbaceous vegetation when wetland drying occurs (Chandler et al., 2017). Of the three most common invertebrate groups found in larval flatwoods salamander stomachs, Isopoda was by far the most abundant in leaf packs, regardless of litter species or habitat type, suggesting that isopods are a plentiful food source in most wetlands. Even though there were no differences in the overall abundance of the three most abundant invertebrate groups predated by flatwoods salamander larvae across habitat types, Cladocera abundance was higher in fire-maintained sections of wetlands, and these small crustaceans are an important part of the diets of small salamander larvae (Whiles et al., 2004). Overall, during a typical cycle of wetland flooding, invertebrate abundance appears unlikely to be a limiting factor for flatwoods salamander larvae but both hydroperiod and the timing of flooding could significantly impact invertebrate community composition and abundance (Gleason & Rooney, 2017).

Compared to many lotic systems, the available information on nutrient cycling, leaf breakdown, and invertebrate communities in ephemeral wetlands embedded within Longleaf Pine forests is minimal. Our study was limited in scope, and future studies could expand on this work in several ways. First, to acquire a more comprehensive understanding of this ecosystem, a complete inventory of aquatic invertebrate species could be conducted in these wetlands, focusing on identifying invertebrates to lower taxonomic levels. Higher taxonomic resolution would allow for a better assessment of the role that specific invertebrates fill in wetland food webs (*e.g.*, Golladay, Taylor & Palik, 1997). Second, the shrub layers that develop in fire-suppressed wetlands are diverse, both in terms of species composition and leaf characteristics. Other common shrub species (*e.g.*, members of the genus *Ilex*) may have different effects on invertebrate communities than those observed in this study. Furthermore, examining the chemical composition of common litter types would shed light on the causal relationships between litter quality and invertebrate abundance. Third, there is a paucity of data on water quality and broader nutrient cycling in pine flatwoods wetlands (but see Sun et al., 2006). Finally, our study only examined leaf breakdown and invertebrate communities across a continuously flooded time period. However, hydrology, especially wetland drying, can have significant effects on these processes (Battle & Golladay, 2007; Gleason & Rooney, 2017), and vegetation community composition can in turn impact wetland hydrology (McLaughlin, Kaplan & Cohen, 2013; Golladay et al., 2021).

## Conclusions

Our study adds to the large body of literature demonstrating the effects of historic and contemporary fire suppression and exclusion on pine ecosystems in the southeastern United States. Our results indicated that vegetation changes associated with a lack of growing-season fires can alter both breakdown and invertebrate communities in wetland systems. Leaf litter inputs into wetlands form the foundation of aquatic food webs and contribute to the overall cycling of nutrients within the wetland. These processes are also linked to the surrounding uplands through annual movements of animals into and out of wetlands in response to flooding events (Smith et al., 2019). Ultimately, management of pine flatwoods wetlands should prioritize maintaining or restoring vegetation structure characteristic of a fire-dependent ecosystem through a combination of mechanical treatments and prescribed fire applied during the growing season or most importantly when wetlands are dry, allowing fire to carry through wetland basins (Gorman, Haas & Himes, 2013).

## Supplemental Information

10.7717/peerj.12534/supp-1 Supplemental Information 1 Leaf litter breakdown and invertebrate count data from leaf packs placed in pine flatwoods wetlands on Eglin Air Force Base, Florida

## Additional Information and Declarations

### Field Study Permissions

The following information was supplied relating to field study approvals (i.e., approving body and any reference numbers): Field experiments and access to field sites were approved by the U.S. Fish and Wildlife Service and Jackson Guard (Eglin Air Force Bases Natural Resources Division) (Cooperative Agreement Number F14AC00068). No permits are required to work with non-listed invertebrates in Florida.

### Data Availability

The following information was supplied regarding data availability: The raw data are available in the Supplemental Files.

## References

[ref-1] Álvarez JA, Bécares E (2006). Seasonal decomposition of *Typha latifolia* in a free-water surface constructed wetland. Ecological Engineering.

[ref-2] Anderson MJ (2006). Distance-based tests for homogeneity of multivariate dispersions. Biometrics.

[ref-3] Anderson MJ, Walsh DC (2013). PERMANOVA, ANOSIM, and the Mantel test in the face of heterogeneous dispersions: what null hypothesis are you testing?. Ecological Monographs.

[ref-4] Bates D, Maechler B. Bolker, M, Walker S (2015). Fitting linear mixed-effects models using lme4. Journal of Statistical Software.

[ref-5] Battle JM, Golladay SW (2001). Hydroperiod influence on breakdown of leaf litter in cypress-gum wetlands. The American Midland Naturalist.

[ref-6] Battle J, Golladay SW (2003). Prescribed fire’s impact on water quality of depressional wetlands in southwestern Georgia. American Midland Naturalist.

[ref-7] Battle JM, Golladay SW (2007). How hydrology, habitat type, and litter quality affect leaf breakdown in wetlands on the Gulf Coastal Plain of Georgia. Wetlands.

[ref-8] Batzer DP, Palik BJ (2007). Variable response by aquatic invertebrates to experimental manipulations of leaf litter input into seasonal woodland ponds. Fundamental and Applied Limnology.

[ref-9] Batzer DP, Wissinger SA (1996). Ecology of insect communities in nontidal wetlands. Annual Review of Entomology.

[ref-10] Becker CG, Rodriguez D, Longo AV, Talaba AL, Zamudio KR (2012). Disease risk in temperate amphibian populations is higher at closed-canopy sites. PLOS ONE.

[ref-11] Beckett DC, Aartila TP, Miller AC (1992). Invertebrate abundance on *Potamogeton nodosus*: effects of plant surface area and condition. Canadian Journal of Zoology.

[ref-12] Benfield EF, Fritz KM, Tiegs SD, Hauer FR, Lamberti GA (2017). Leaf litter breakdown. Methods in stream ecology. Volume 2: ecosystem function.

[ref-13] Bishop DC, Haas CA (2005). Burning trends and potential negative impacts on flatwoods salamanders. Natural Areas Journal.

[ref-14] Bisson PA, Rieman BE, Luce C, Hessburrg PF, Lee DC, Kershner JL, Reeves GH, Gresswell RE (2003). Fire and aquatic ecosystems of the western USA: current knowledge and key questions. Forest Ecology and Management.

[ref-15] Bixby RJ, Cooper SD, Gresswell RE, Brown LE, Dahm CN, Dwire KA (2015). Fire effects on aquatic ecosystems: an assessment of the current state of the science. Freshwater Science.

[ref-16] Brockway DG, Lewis CE (1997). Long-term effects of dormant-season prescribed fire on plant community diversity, structure and productivity in a longleaf pine wiregrass ecosystem. Forest Ecology and Management.

[ref-17] Brooks GC, Smith JA, Frimpong EA, Gorman TA, Chandler HC, Haas CA (2019). Indirect connectivity estimates of amphibian breeding wetlands from spatially explicit occupancy models. Aquatic Conservation: Marine and Freshwater Ecosystems.

[ref-18] Butz RJ (2009). Traditional fire management: historical fire regimes and land use change in pastoral East Africa. International Journal of Wildland Fire.

[ref-19] Carlson PC, Tanner GW, Wood JM, Humphrey SR (1993). Fire in key deer habitat improves browse, prevents succession, and preserves endemic herbs. The Journal of Wildlife Management.

[ref-20] Chandler HC (2015). The effects of climate change and long-term fire suppression on ephemeral pond communities in the southeastern United States. Thesis.

[ref-21] Chandler HC, Haas CA, Gorman TA (2015). The effects of habitat structure on winter aquatic invertebrate and amphibian communities in pine flatwoods wetlands. Wetlands.

[ref-22] Chandler HC, McLaughlin DL, Gorman TA, McGuire KJ, Feaga JB, Haas CA (2017). Drying rates of ephemeral wetlands: implications for breeding amphibians. Wetlands.

[ref-23] Clarke KR (1993). Non-parametric multivariate analysis of changes in community structure. Australian Journal of Ecology.

[ref-24] Cohen MJ, Creed IF, Alexander L, Basu NB, A. Calhoun JK, Craft C, D’Amico E, De Keyser E, Fowler L, Golden HE, Jawitz JW, Kalla P, Kirkman LK, Lane CR, Lang M, Leibowitz SG, Lewis DB, Marton J, McLaughlin DL, Mushet DM, Raanan-Kiperwas H, Rains MC, Smith L, Walls SC (2016). Do geographically isolated wetlands influence landscape functions?. Proceedings of the National Academy of Sciences of the United States of America.

[ref-25] Conway CJ, Nadeau CP, Piest L (2010). Fire helps restore natural disturbance regime to benefit rare and endangered marsh birds endemic to the Colorado River. Ecological Applications.

[ref-26] Daubenmire RF (1959). A canopy-cover method of vegetational analysis. Northwest Science.

[ref-27] De Szalay FA, Resh VH (1997). Responses of wetland invertebrates and plants important in waterfowl diets to burning and mowing of emergent vegetation. Wetlands.

[ref-28] Erwin KJ, Chandler HC, Palis JG, Gorman TA, Haas CA (2016). Herpetofaunal communities in ephemeral wetlands embedded within longleaf pine flatwoods of the Gulf Coastal Plain. Southeastern Naturalist.

[ref-29] Fazi S, Rossi L (2000). Effects of macro-detritivores density on leaf detritus processing rate: a macrocosm experiment. Hydrobiologia.

[ref-30] Frost CC, Hermann SM (1993). Four centuries of changing landscape patterns in the longleaf pine ecosystem. Proceedings of the Tall timbers fire ecology conference, (18) the longleaf pine ecosystem: ecology, restoration and management.

[ref-31] Fuell AK, Entrekin SA, Owen GS, Owen SK (2013). Drivers of leaf decomposition in two wetland types in the Arkansas River Valley, U.S.A.. Wetlands.

[ref-32] Gilliam FS, Platt WJ (1999). Effects of long-term fire exclusion on tree species composition and stand structure in an old-growth *Pinus palustris* (Longleaf pine) forest. Plant Ecology.

[ref-33] Gingerich RT, Panaccione DG, Anderson JT (2015). The role of fungi and invertebrates in litter decomposition in mitigated and reference wetlands. Limnologica.

[ref-34] Gleason JE, Rooney RC (2017). Pond permanence is a key determinant of aquatic macroinvertebrate community structure in wetlands. Freshwater Biology.

[ref-35] Glitzenstein JS, Streng DR, Wade DD (2003). Fire frequency effects on Longleaf Pine (*Pinus palustris* P. Miller) vegetation in South Carolina and Northeast Florida, USA. Natural Areas Journal.

[ref-36] Golladay SW, Clayton BA, Brantley ST, Smith CR, Qi J, Hicks DW (2021). Forest restoration increases isolated wetland hydroperiod: a long-term case study. Ecosphere.

[ref-37] Golladay SW, Taylor BW, Palik BJ (1997). Invertebrate communities of forested limesink wetlands in southwest Georgia, USA: habitat use and influence of extended inundation. Wetlands.

[ref-38] Gorman TA, Haas CA, Bishop DC (2009). Factors related to occupancy of breeding wetlands by flatwoods salamander larvae. Wetlands.

[ref-39] Gorman TA, Haas CA, Himes JG (2013). Evaluating methods to restore amphibian habitat in fire-suppressed pine flatwoods wetlands. Fire Ecology.

[ref-40] Gorman TA, Powell SD, Jones KC, Haas CA (2014). Microhabitat characteristics of egg deposition sites used by reticulated flatwoods salamanders. Herpetological Conservation and Biology.

[ref-41] Henderson JP (2006). Dendroclimatological analysis and fire history of Longleaf Pine (*Pinus palustris* Mill.) in the Atlantic and Gulf Coastal Plain. Dissertation, University of Tennessee.

[ref-42] Hinman SE, Brewer JS (2007). Responses of two frequently-burned wet pine savannas to an extended period without fire. The Journal of the Torrey Botanical Society.

[ref-43] Hisabae M, Sone S, Inoue M (2010). Breakdown and macroinvertebrate colonization of needle and leaf litter in conifer plantation streams in Shikoku, southwestern Japan. Journal of Forest Research.

[ref-44] Hornung JP, Foote AL (2006). Aquatic invertebrate responses to fish presence and vegetation complexity in western boreal wetlands, with implications for waterbird productivity. Wetlands.

[ref-45] Jeffries M (1993). Invertebrate colonization of artificial pondweeds of differing fractal dimension. Oikos.

[ref-46] Johnstone JF, Allen CD, Franklin JF, Frelich LE, Harvey BJ, Higuera PE, Mack MC, Meentemeyer RK, Metz MR, Perry LWG, Schoennagel T, Turner MG (2016). Changing disturbance regimes, ecological memory, and forest resilience. Frontiers in Ecology and the Environment.

[ref-47] Kirkman LK (1995). Impacts of fire and hydrological regimes on vegetation in depression wetlands of southeastern USA. Fire in wetlands: a management perspective. Proceedings of the tall timbers fire ecology conference.

[ref-48] Kirkman LK, Golladay SW, Laclaire L, Sutter RD (1999). Biodiversity in southeastern, seasonally ponded, isolated wetlands: management and policy perspectives for research and conservation. Journal of the North American Benthological Society.

[ref-49] Lenth RV (2021). emmeans: estimated marginal means, aka Least-squares means. https://CRAN.R-project.org/package=emmeans.

[ref-50] Leroy CJ, Marks JC (2006). Litter quality, stream characteristics and litter diversity influence decomposition rates and macroinvertebrates. Freshwater Biology.

[ref-51] Long JN (2009). Emulating natural disturbance regimes as a basis for forest management: A North American view. Forest Ecology and Management.

[ref-52] Maloney DC, Lamberti GA (1995). Rapid decomposition of summer -input leaves in a northern Michigan stream. The American Midland Naturalist.

[ref-53] McInerney PJ, Stoffels RJ, Shackleton ME, Davey CD (2017). Flooding drives a macroinvertebrate biomass boom in ephemeral floodplain wetlands. Freshwater Science.

[ref-54] McLaughlin DL, Kaplan DA, Cohen MJ (2013). Managing forests for increased regional water yield in the southeastern U.S. Coastal Plain. Journal of American Water Resource Association.

[ref-55] Morgan P, Hardy CC, Swetnam TW, Rollins MG, Long DG (2001). Mapping fire regimes across time and space: understanding coarse and fine-scale fire patterns. International Journal of Wildland Fire.

[ref-56] Mouillot F, Field CB (2005). Fire history and the global carbon budget: A 1° ×1° fire history reconstruction for the 20th century. Global Change Biology.

[ref-57] Mulhouse JM, Steven DD, Lide RF, Sharitz RR (2005). Effects of dominant species on vegetation change in Carolina bay wetlands following a multi-year drought. Journal of the Torrey Botanical Society.

[ref-58] Murkin HR, Wrubleski DA (1988). Aquatic invertebrates of freshwater wetlands: function and ecology. The ecology and management of wetlands.

[ref-59] Newman EA (2019). Disturbance ecology in the Anthropocene. Frontiers in Ecology and Evolution.

[ref-60] Oksanen J, Blanchet FG, Friendly M, Kindt R, Legendre P, McGlinn D, Minchin PR, O’Hara RB, Simpson GL, Solymos P, Stevens HHM, Szoecs E, Wagner H (2019). vegan: community Ecology Package. https://CRAN.R-project.org/package=vegan.

[ref-61] Palis JG (1996). Flatwoods salamander (*Ambystoma cingulatum* Cope). Natural Areas Journal.

[ref-62] Petersen RC, Cummins KW (1974). Leaf processing in a woodland stream. Freshwater Biology.

[ref-63] Peterson DW, Reich PB (2008). Fire frequency and tree canopy structure influence plant species diversity in a forest-grassland ecotone. Plant Ecology.

[ref-64] Polyakova O, Billor N (2007). Impact of deciduous tree species on litterfall quality, decomposition rates and nutrient circulation in pine stands. Forest Ecology and Management.

[ref-65] Powell TL, Starr KL, Clark TA, Martin G, Gholz HL (2005). Ecosystem and understory water and energy exchange for a mature, naturally regenerated pine flatwoods forest in north Florida. Canadian Journal of Forest Research.

[ref-66] R Core Team (2020). R: a language and environment for statistical computing. https://www.R-project.org/.

[ref-67] Sakai M, Fukushima K, Naito RS, Natuhara Y, Kato M (2016). Coniferous needle litter acts as a stable food resource for stream detritivores. Hydrobiologia.

[ref-68] Sekerak CM, Tanner GW, Palis JG (1996). Ecology of flatwoods salamander larvae in breeding ponds in Apalachicola National Forest. Proceedings of the Annual Conference of the Southeastern Association of Fish and Wildlife Agencies.

[ref-69] Smith LL, Subalusky AL, Atkinson CL, Earl JE, Mushet DM, Scott DE, Lance SL, Johnson SA (2019). Biological connectivity of seasonally ponded wetlands across spatial and temporal scales. Journal of the American Water Resources Association.

[ref-70] Stambaugh MC, Guyette RP, Marshall JM (2011). Longleaf pine (*Pinus palustris* Mill.) fire scars reveal new details of a frequent fire regime. Journal of Vegetation Science.

[ref-71] Stoler AB, Relyea RA (2016). Leaf litter species identity alters the structure of pond communities. Oikos.

[ref-72] Sun G, Li C, Trettin CC, Lu J, McNulty SG (2006). Simulating the biogeochemical cycles in cypress wetland-pine upland ecosystems at a landscape scale with the wetland-DNDC model.

[ref-73] Tiegs SD, Peter FD, Robinson CT, Uehlinger U, Gessner MO (2008). Leaf decomposition and invertebrate colonization responses to manipulated litter quantity in streams. Journal of the North American Benthological Society.

[ref-74] Tietema A, Wessel WW (1993). Microbial activity and leaching during initial oak leaf litter decomposition. Biology and Fertility of Soils.

[ref-75] Tiner RW (2003). Geographically isolated wetlands of the United States. Wetlands.

[ref-76] Townsend CR, Scarsbrook MR (1997). The intermediate disturbance hypothesis, refugia, and biodiversity in streams. Limnology and Oceanography.

[ref-77] Turner MG (2010). Disturbance and landscape dynamics in a changing world. Ecology.

[ref-78] U. S. Fish and Wildlife Service (2009). Endangered and threatened wildlife and plants; determination of endangered status for Reticulated Flatwoods Salamander; designation of critical habitat for Frosted Flatwoods Salamander and Reticulated Flatwoods Salamander. Federal Register.

[ref-79] Watts AC (2013). Organic soil combustion in cypress swamps: moisture effects and landscape implications for carbon release. Forest Ecology and Management.

[ref-80] Wendt A, Haas CA, Gorman TA, Roberts JH (2021). Metapopulation genetics of endangered reticulated flatwoods salamanders (*Ambystoma bishopi*) in a dynamic and fragmented landscape. Conservation Genetics.

[ref-81] Werner EE, Glennemeier KS (1999). Influence of forest canopy cover on the breeding pond distributions of several amphibian species. Copeia.

[ref-82] Whiles MR, Jensen JB, Palis JG, Dyer WG (2004). Diets of larval Flatwoods Salamanders, *Ambystoma cingulatum*, from Florida and South Carolina. Journal of Herpetology.

[ref-83] White PS, Jentsch A (2001). The search for generality in studies of disturbance and ecosystem dynamics. Progress in Botany.

[ref-84] White PS, Pickett STA, Pickett STA, White PS (1985). Natural disturbance and patch dynamics:an introduction. The ecology of natural disturbance and patch dynamics.

[ref-85] Zimmer KD, Hanson MA, Butler MG, Duffy WG (2001). Size distribution of aquatic invertebrates in two prairie wetlands, with and without fish, with implications for community production. Freshwater Biology.

